# Increased postpartum haemorrhage, the possible relation with serotonergic and other psychopharmacological drugs: a matched cohort study

**DOI:** 10.1186/s12884-017-1334-4

**Published:** 2017-06-02

**Authors:** Hanna M. Heller, Anita C. J. Ravelli, Andrea H. L. Bruning, Christianne J. M. de Groot, Fedde Scheele, Maria G. van Pampus, Adriaan Honig

**Affiliations:** 1 Department of Hospital Psychiatry, VU University Medical Centre, De Boelelaan 1117, 1081 HV Amsterdam, The Netherlands; 2 Department of Medical Informatics, Academic Medical Centre, University of Amsterdam, Meibergdreef 9, 1105 AZ Amsterdam, The Netherlands; 3 Department of Pediatric Infectious Diseases, Emma Children’s Hospital, Academic Medical Centre, University of Amsterdam, Meibergdreef 9, 1105 AZ Amsterdam, The Netherlands; 4 Department of Obstetrics and Gynaecology, VU University Medical Centre, De Boelelaan 1117, 1081 HV Amsterdam, The Netherlands; 5 Department of Obstetrics and Gynaecology, OLVG Hospital (West), Jan Tooropstraat 164, 1061 AE Amsterdam, The Netherlands; 6 Department of Obstetrics and Gynaecology, VU University Medical Centre, De Boelelaan 1117, 1081 HV Amsterdam, The Netherlands; 7 Department of Obstetrics and Gynaecology, OLVG Hospital (East), Oosterpark 9, 1091 AC Amsterdam, The Netherlands; 8 Department of Hospital Psychiatry, OLVG Hospital (West), Jan Tooropstraat 164, 1061 AE Amsterdam, The Netherlands; 9 Department of Hospital Psychiatry, VU University Medical Centre, De Boelelaan 1117, 1081 HV Amsterdam, The Netherlands

**Keywords:** Postpartum haemorrhage, Antidepressants, Serotonin, Postpartum blood loss, Pregnancy, Selective serotonin reuptake inhibitors, Psychopharmacological medication

## Abstract

**Background:**

Postpartum haemorrhage is a major obstetric risk worldwide. Therefore risk factors need to be investigated to control for this serious complication. A recent systematic review and meta-analysis revealed that the use of both serotonergic and non-serotonergic antidepressants in pregnancy are associated with a higher risk of postpartum haemorrhage. However, use of antidepressants in pregnancy is often necessary because untreated depression in pregnancy is associated with adverse maternal and neonatal outcome, such as postpartum depression, preterm birth and dysmaturity. Therefore it is of utmost importance to unravel the possible association between postpartum haemorrhage and the use of serotonergic and other psychopharmacological medication during pregnancy.

**Methods:**

We performed a matched cohort observational study consecutively including all pregnant women using serotonergic medication (*n* = 578) or other psychopharmacological medication (*n* = 50) visiting two teaching hospitals in Amsterdam between 2010 and 2014. The incidence of postpartum haemorrhage in women using serotonergic medication or other psychopharmacological medication was compared with the incidence of postpartum haemorrhage in 641,364 pregnant women not using psychiatric medication selected from the database of the Netherlands Perinatal Registry foundation (Perined). Matching took place 1:5 for nine factors, i.e., parity, maternal age, ethnicity, socioeconomic status, macrosomia, gestational duration, history of postpartum haemorrhage, labour induction and hypertensive disorder.

**Results:**

Postpartum haemorrhage occurred in 9.7% of the women using serotonergic medication. In the matched controls this was 6.6% (*p* = 0.01). The adjusted odds ratio (aOR) before matching was 1.6 (95% CI 1.2–2.1) and after matching 1.5 (95% CI 1.1–2.1). Among the women using other psychopharmacological medication, the incidence of postpartum haemorrhage before matching was 12.0% versus 6.1% (*p* = 0.08) with OR 2.1 (95% CI 0.9–4.9), and after matching 12.1% versus 4.4% (*p* = 0.03) with aOR of 3.3 (95% CI 1.1–9.8).

**Conclusions:**

Pregnant women using serotonergic medication have an increased risk of postpartum haemorrhage, but this high risk is also seen in pregnant women using other psychopharmacological medication. We suggest that this higher risk of postpartum haemorrhage could not only be explained by serotonin, but also by other mechanisms. An additional explanation could be the underlying psychiatric disorder.

## Background

Postpartum haemorrhage (PPH) is a major obstetric risk worldwide, with an increasing incidence in developed countries [1, 2]. In the Netherlands the overall incidence of PPH defined as blood loss of more than 1000 ml was around 6% in 2014 [3]. Despite the growing knowledge about risk factors, we are still unable to fully explain the increasing incidence of this obstetric complication. Recent studies suggest that the use of serotonergic antidepressants in pregnancy might be associated with a higher risk of PPH [4–8].

Several studies have investigated the association of serotonergic antidepressant exposure during pregnancy and PPH, with varying outcomes [4]. Some studies concluded that pregnant women using serotonergic antidepressants have an almost two times higher risk of PPH [5–9] whilst others reported no increased risk [10–13]. A recent systematic review and meta-analysis revealed that not only SSRI’s, but also non serotonergic antidepressants are associated with a higher risk on PPH [14].

Treatment with antidepressants is often discontinued or not started or resumed during pregnancy because of possible foetal and obstetric complications [15]. On the other hand, untreated depression in pregnancy is associated with adverse neonatal outcome, such as an increased risk of prematurity, dysmaturity, decreased breastfeeding initiation [16, 17], increased risk of operative delivery [18–20], increased risk of caesarean delivery or rupture of the anal sphincter [21], increased risk of postpartum depression [22, 23] and affective and behavioural consequences for the offspring [24, 25]. Some of these complications are associated with postpartum haemorrhage. Therefore, the use of antidepressants in pregnancy mostly outweighs its potential risks; its use is increasing in more highly developed countries [26–28]. This underscores the need to investigate the effectiveness and safety of treatment options for depression in pregnancy and postpartum.

The most frequently prescribed antidepressants, the serotonin reuptake inhibitors (SSRIs), are estimated to be used by 2 to 3% of the pregnant women in Europe and by 10% in North America [15]. A frequently proposed hypothesis for the aetiology of PPH in these women is that the use of serotonergic medication, more specifically the serotonergic action of these drugs on platelets, causes diminished blood clotting [29].

However, the varying incidence of PPH in these women may also relate to confounding due to the large number of other factors that contribute to this complication. It is therefore important to properly adjust for these confounding factors [4]. The first aim of our study was to investigate the role of serotonergic medication on the development of PPH reducing as much as possible the effect of potential confounders by performing a matched paired cohort analysis.

Our secondary aim was to determine the effect of other psychopharmacological medication, such as lithium and antipsychotics on the development of PPH. However, we didn’t expect antipsychotics to increase PPH, because most studies show that the use of especially second generation antipsychotics leads to an increased risk of platelet aggregation. [30]. Some other psychopharmacological drugs are supposed to have also a kind of serotonergic action including contributing to the serotonin syndrome [31] but are not the affecting the blood clotting in the same way.

Based on previous studies, we hypothesized that the risk of PPH is higher for serotonergic drugs than for no drugs or for other psychopharmacological medication. This question about the contribution of serotonergic drugs to the development of PPH is of the utmost importance since use in pregnancy has increased and better knowledge of outcomes can help pregnant women and clinicians make more informed decisions concerning care.

## Methods

### Setting, population and study participants

We consecutively included all women in two hospitals, Sint Lucas Andreas Hospital and Onze Lieve Vrouwe Gasthuis, in Amsterdam, the Netherlands, between 2007 and 2014 who used psychopharmacological medication during pregnancy. In the Netherlands most pregnant women receive prenatal care from a midwife residing in or outside the hospital. Only women with (expected) complications during pregnancy or delivery are seen by a gynaecologist. Since taking psychotropic medication may cause for example withdrawal symptoms in the baby or postpartum haemorrhage in the mother this can be a (sole) reason for referral to a gynaecologist.

The data from their visits to the hospitals and their deliveries were extracted from the patient charts. The study was approved by the research and ethics board of both hospitals (registration number 14–096).

The cohort was defined as consisting of single pregnancies of women who used psychopharmacological medication in the third trimester. The first cohort group used serotonergic antidepressants (SSRIs, TCAs or SNRIs) with or without other psychopharmacological medication (cohort 1) and the second cohort group only used other psychopharmacological medication (antipsychotics, lithium) but no antidepressants, except for bupropion(cohort 2), all in the third trimester. Bupropion was classified in the non-serotonergic group since its action is related to the inhibition of dopamine and noradrenaline reuptake. All cohort members delivered from 32.0 weeks onwards.

### Study design

We conducted a matched cohort study consisting of either pregnancies of women taking serotonergic medication or pregnancies of women taking other psychopharmacological medication. We matched these women to pregnancies of women without documented psychiatric problems and measured the outcome PPH, since data on the use of psychiatric medication in the control population were not available.

### Controls

To identify matched controls, we used the linked national perinatal database of the Netherlands Perinatal Registry foundation (PRN or named PERINED since 2016; PERINED approvalnr. 15.34) The PERINED registry covers 96% of all deliveries in the Netherlands and contains anonymous information on pregnancies, deliveries ≥ 22 weeks of gestation and readmission of the child until 28 days after birth [32]. Because the definition of the PERINED database variables changed from 2010 onwards, we used the data set of the recent years 2010–2014.

Exclusion criteria for controls were documented psychiatric problems, delivery in either of the two hospitals or the primary care facility where the cohort members delivered, and women with a length of gestation of less than 32 weeks.

We matched for parity (nulliparity vs multiparity), maternal age (<35, ≥35 years), ethnicity (Caucasian, non-Caucasian), socioeconomic status (low, middle/high), macrosomia (birth weight above 90^th^ percentile adjusted for gestational age and parity), gestational duration (≤37 or >37 weeks), history of PPH (yes/no), induction of labour and hypertensive disorder (yes/no) to make the characteristics of the “type of woman” who used serotonergic or other medication comparable to the controls. We selected 5 controls for each cohort member and matched on equality or nearest neighbour.

In the Netherlands the socioeconomic status (SES) is determined by the Netherlands Institute for Social Research based on the 4-digit zip code, and the score is divided into low SES versus mid/high. The zip code is used nationwide to classify neighbourhoods for example as disadvantaged based om population density, educational level and percentage of low income [33]. The 90^th^ percentile is based on the Dutch reference curve for birthweight by gestational age, parity, ethnicity and gender [34].

The matching factors were based on the 2009 guidelines of the Royal College of Obstetricians and Gynaecologists (revised in 2011), identified factors in literature, clinical reasoning and availability in both databases.

### Primary outcome measurements

Outcome was defined as PPH with postpartum blood loss exceeding 1000 ml.

### Statistical analysis

At first, in the ‘unmatched’ cohort, we used traditional analysing techniques (i.e., crude and adjusted logistic regression) to control for confounders in the women who used serotonergic medication and compared them with the controls. Univariate analyses of the baseline characteristics were performed with a chi-square test for categorical variables. Next we matched on the nine factors and repeated the analysis. Maternal and pregnancy baseline characteristics of the women who used serotonergic antidepressants and all the controls were described.

Secondarily, we repeated the analysis, the matching and the analysis after matching for the patients who only used other (non-serotonergic) psychopharmacological medication and compared these with the controls. To estimate the influence of the serotonergic and other psychopharmacological medication on PPH, univariate and multivariate logistic regression analyses were performed both before and after matching. In the adjusted logistic regression analysis, the same nine matching variables were used, supplemented with year of birth of the child.

We could not match for intermediate variables in the causal path of postpartum haemorrhage. The intermediate factors were mode of delivery (operative vaginal delivery or delivery by emergency caesarean section), episiotomy, perineal laceration (defined as third- and fourth-degree tears “total rupture”) and retained placenta. Therefore, in addition to the matching, we performed a stratified analysis only for the cohort using serotonergic medication due to the small numbers in the cohort of women using other psychopharmacological medication.

All statistical tests were two-sided, and a *p* value of 0.05 was chosen as the threshold for statistical significance. Data were analysed using SAS software version 9.3 (SAS institute Inc., Cary, NC, USA). The matching was analysed with R statistical software version 3.2.0 (T Foundation for Statistical computing, Vienna, Austria) using the Matchit package.

## Results

In total, 628 singleton pregnancies in women using psychopharmacological medication during the third trimester of pregnancy were included. There were 578 pregnancies in cohort 1 (women who used serotonergic medication) and 50 pregnancies in cohort 2 (women who used other psychopharmacological drugs). Of cohort 2 group, 27 women used antipsychotics (haloperidol, quetiapine, olanzapine, clozapine or flupentixol), eight used a mood stabiliser (lithium or sodium valproate), five used antipsychotics and also a mood stabiliser, two used antipsychotics and also a benzodiazepine, three used only benzodiazepines, one used an antipsychotic and bupropion, and five used only bupropion. 86% of these used monotherapy only. There were 641,364 controls.

### Women with serotonergic medication (cohort 1 group)

Women in cohort 1 were more likely to be older than 35 years and to have a low socioeconomic status (SES). They were less likely to have Caucasian ethnicity. This group also had a higher percentage of women with a previous pregnancy complicated by premature birth and/or PPH (Table 1). No differences were found between the two groups for parity, induction of labour, maternal hypertension and macrosomia in the index pregnancy.

**Table 1:** Baseline characteristics and the outcomes of the 578 women using serotonergic medication and the controls before and after matching

	Before matching					After matching with 5 controls				
	Women with SRmed		Controls (Perined)			Women with SRmed			Controls (Perined)	
	(*n* = 578)	%	(*n* = 641,364)	%	*p*-value	(*n* = 578)	%	(*n* = 2890)	%	*p*-value
Characteristics										
Nulliparity	258	44.6%	289,190	45.1%	NS	258	44.6%	1290	44.6%	NS
Caucasian ethnicity	351	60.7%	522,141	81.4%	<0.0001	351	60.7%	1750	60.6%	NS
Low socioeconomic status	286	49.5%	155,183	24.2%	<0.0001	286	49.5%	1429	49.4%	NS
Maternal age > = 35 years	195	33.7%	131,584	20.5%	<0.0001	195	33.7%	971	33.6%	NS
Previous PPH	16	2.8%	6782	1.1%	<0.0001	16	2.8%	80	2.8%	NS
Induction of labour	135	23.4%	135,251	21.1%	NS	135	23.4%	674	23.3%	NS
Hypertension	47	8.1%	52,763	8.2%	NS	47	8.1%	230	8.0%	NS
Macrosomia P90	59	10.2%	65,522	10.2%	NS	59	10.2%	290	10.0%	NS
Gestational duration <37 weeks	43	7.4%	34,322	5.4%	0.0258	43	7.4%	216	7.5%	NS
Outcome										
PPH (>1000 ml)	56	9.7%	39,171	6.1%	0.0003	56	9.7%	191	6.6%	0.009

SRmed: serotonergic medication

Before matching, PPH was more frequent in cohort 1 than among the 641,364 controls (9.7% versus 6.1%, *p* <0.0003). The adjusted odds ratio (aOR) was 1.6 (95% CI 1.2–2.1) (Table 2).

**Table 2:** Postpartum haemorrhage after use of serotonergic medication (*N* = 578) versus controls before and after matching

	Crude Odds		Adjusted Odds^a^	
	95% C.I.		95% C.I.	
Outcome before matching				
postpartum haemorrhage	1.7	1.3–2.2	1.6	1.2–2.1
Outcome after matching				
postpartum haemorrhage	1.5	1.1–2.1	1.5	1.1–2.1

^a^Adjusted for parity, maternal age, ethnicity, socio-economic status, previous postpartum haemorrhage, hypertension, macrosomia, induction of labour, year of birth and prematurity

After matching, cohort 1 was compared with 2890 controls and the differences in the baseline characteristics disappeared (all *p*-values were non-significant). In cohort 1, the percentage of PPH was significantly higher than controls, 9.7% versus 6.6% (*p* = 0.0086) (Table 1). The aOR was also significantly increased to 1.5 (95% CI 1.1–2.1) (Table 2).

### Women using other psychopharmacological medication (cohort 2 group)

First, we compared cohort 2 with the 641,364 controls (Table 3).

**Table 3:** Baseline characteristics and the outcomes of the 50 women using other psychopharmacological medication and the controls before and after matching

	Before matching					After matching with 5 controls				
	other ps.drugs		Controls (Perined)			other ps.drugs		Controls (Perined)		
	(*n* = 50)	%	(*n* = 641,364)	%	*p*-value	*n* = 50	%	*n* = 250	%	*p*-value
Characteristics										
Nulliparity	29	58.0%	289,190	45.1%	0.0666	29	58.0%	145	58.0%	NS
Caucasian ethnicity	26	52.0%	522,141	81.4%	<0.0001	26	52.0%	130	52.0%	NS
Low socioeconomic status	23	46.0%	155,183	24.2%	0.0003	23	46.0%	115	46.0%	NS
Maternal age > = 35 years	16	32.0%	131,584	20.5%	0.044	16	32.0%	80	32.0%	NS
Previous PPH	0	0.0%	6782	1.1%	NS	0	0.0%	0	0.0%	NS
Induction of labour	15	30.0%	135,251	21.1%	NS	15	30.0%	75	30.0%	NS
Hypertension	4	8.0%	52,763	8.2%	NS	4	8.0%	20	8.0%	NS
Macrosomia P90	6	12.0%	65,522	10.2%	NS	6	12.0%	30	12.0%	NS
Gestational duration <37 weeks	5	10.0%	34,322	5.4%	NS	5	10.0%	25	10.0%	NS
Outcome										
PPH (>1000 ml)	6	12.0%	39,171	6.1%	0.0819	6	12.0%	11	4.4%	0.0339

ps.drugs: other psycofarmacological drugs

Before matching, cohort 2 comprised more nulliparous women and more women with non-Caucasian ethnicity, low SES and increased maternal age than the control group. The percentage of PPH in cohort 2 did not differ significantly from the percentage in the controls (12.0% versus 6.1%, (*p* = 0.082). The adjusted odds ratio did not significantly increase: aOR 2.1 (95% CI 0.9–4.9) (Table 4).

**Table 4:** Postpartum haemorrhage after use of other psychopharmacological drugs (*n* = 50) before and after matching

	Crude Odds		Adjusted Odds^a^	
	95% C.I.		95% C.I.	
Outcome before matching				
Postpartum haemorrhage	2.1	0.9–4.9	2.1	0.9–4.9
Outcome after matching				
Postpartum haemorrhage	3.0	1.0–8.4	3.3	1.1–9.8

^a^Adjusted for parity, maternal age, ethnicity, socio-economic status, previous postpartum haemorrhage, hypertension, macrosomia, induction of labour, year of birth and prematurity

After matching, cohort 2 was comparable to 250 controls, and the incidence of PPH among the target population with other psychopharmacological drugs was significantly higher than among these comparable controls (12.1% versus 4.4% (*p* = 0.034). The adjusted odds ratio was significantly higher: 3.3 (95% CI 1.1–9.8) (Tables 3 and 4).

### Stratified analyses

We performed a stratified analysis for intermediate factors in cohort 1 with a PPH of 9.7% and the 2890 controls with a PPH of 6.6%. In cohort 1, PPH was more common among women with operative vaginal deliveries (15.7% versus 7.5%; *p* = 0.0083) than in controls with operative vaginal deliveries. PPH was also more common among women in cohort 1 who delivered via emergency caesarean delivery (19.6% versus 5.7%;*p* = 0.0009).

However, in cohort 1, women with a perineal laceration had a lower incidence of PPH than controls with a perineal laceration (3.8% versus 12.9%, *p* = 0.0266). Among women with retained placentas, the risk of PPH was very high, but this risk was lower in women of cohort 1 compared to the controls (36.7% versus 62.6%; *p* = 0.0034).

## Discussion

### Main findings

In our study we tried to unravel the influence the role of serotonergic medication on the development of PPH reducing as much as possible the effect of potential confounders by performing a matched paired cohort analysis. Secondary we tried to investigate the influence of other psychopharmacological medication on the development of PPH.

Our findings of a higher prevalence of PPH among women using serotonergic medication in the third trimester of pregnancy is in line with the hypothesis we based on previous studies.

On the other hand this prevalence was also increased in women using other psychopharmacological medication. In our population of women using medication with a serotonergic action, we found a 1.5-fold higher percentage of PPH than in a matched control population of women who did not take any psychopharmacological medication. In the group of women using other psychopharmacological medication, this percentage was almost 3 times larger than in the matched control population. In both groups of women using any psychopharmacological medication, the adjusted odds ratios of PPH were significantly higher than in the control group (1.5 resp. 3.3, Tables 2 and 4).

### Strengths and limitations

Several strengths and limitations of our study design need to be addressed. The first strength is that we compared the incidence of PPH both in women using serotonergic antidepressants and in women using other psychopharmacological medication to the incidence in women not using any psychopharmacological medication. Secondly, by using a match cohort study we have matched our study population for nine possible confounding variables. In this way we could, despite our small study population, minimize the influence of well-established maternal and pregnancy confounders. Having 5 controls instead of 3 or 1 per participant increased the sample size [35]. A match cohort study can minimize observed measured differences in characteristics among the participants and the controls.

However they cannot rule out unmeasured and unknown factors. Therefore, a limitation is that we had to leave out two well established matching variables, BMI and smoking, since these variables unfortunately were not available in the national PERINED databases. However, the incidence of obesity and smoking in our population was low, 5% of the pregnant women smoked more than 1 cigarette a day and 1,5% smoked more than 10 cigarettes a day. Obesity, defined as BMI above 30 kg/m^2^ occurred in 3.3% of our study population. These data suggest that these confounders probably did not have a considerable influence on our results.

A second limitation is that in the serotonergic cohort some of the matched factors could be also considered as intermediate factors, like hypertensive disorders in pregnancy, foetal growth retardation and preterm birth. Therefore we repeated the matching with only five factors and performed a conditional analysis for this group. The adjusted odds ratio of the women using serotoninergic drugs was 1.7 (CI 1.2–1.3) which is even higher than in the first matching and analysis. We performed the matching with five factors and conditional analysis in the other psychopharmacological cohort (*n* = 50), and the risk was also increased (12% versus 6%) but no longer significant i.e., 1.9 (CI 0.7–5.4). This can be explained through the fact that the numbers of PPH cases are small in this cohort. Another explanation could be that the previous mentioned intermediate factors in the serotonergic cohort are not in the same way applicable to the cohort of women using other psychopharmacological medication [36–38].

A third limitation is that the PERINED database does not have information on the actual medication of women during pregnancy nor on depression during pregnancy. Nevertheless, we excluded all women with records of psychological problems or diseases in the PERINED database, but we cannot completely rule out that some women among the controls may also have used serotonergic or other psychopharmacological medication. On the other hand, this limitation only means that we may have underestimated the effects found in this study. A third limitation is that cohort 2 was relatively small, which made it difficult to perform a stratified analysis in this group.

### Interpretation

Our finding that the use of serotoninergic drugs increases the risk of PPH is in agreement with at least five previous studies [5–9]. Some of these studies compared the risk of PPH between women using the medication in different stages of pregnancy [9], and other studies examined women with a psychiatric illness without medication [5]. These approaches do not take into account the potential role of the severity of the underlying psychiatric disorder, since taking women with psychiatric complaints but not using medication as controls probably implies leaving out the more severely ill. Also in those investigations, the severity of psychiatric disorders rather than medication use could be the risk factor related to PPH, since women with stress due to psychiatric problems may be more prone to prolonged labour [21, 39]. Prolonged labour in itself constitutes a risk factor for PPH [40], but also for delivery by emergency caesarean section and for operative vaginal delivery [18, 19], and both types of delivery also contribute to the development of PPH.

A second explanation for other psychopharmacological medication generating a higher incidence of PPH could be that even though this medication does not directly influence serotoninergic action, it might affect other neurotransmitters and thus create a shift in the neurotransmitter balance, which may indirectly affect the platelet function.

## Conclusion

Our study sheds a new light on the possible influence of psychopharmacological medication on postpartum haemorrhage. The hypothesis that the serotonergic action of antidepressant medication plays a main role in the development of PPH probably does not constitute the complete explanation. The results of our study suggest that serotonergic medication but also other psychopharmacological medication during pregnancy can result in a higher risk of PPH. However, it could be plausible that the acting mechanism is not only the medication, but also the underlying disease.

Future research should focus on blood loss postpartum in women using all types of psychopharmacological medication compared to women with equally serious psychiatric illness who do not use any of this medication during pregnancy.

## Abbreviations

PPH: Postpartum haemorrhage
SES: Social economic status
BMI: Body mass index
PERINED: Netherlands perinatal registry foundation
SSRIs: Selective serotonin reuptake inhibitors
TCAs: Tricyclic antidepressants
SNRIs: Serotonin and noradrenalin reuptake inhibitors
aOR: Adjusted odds ratio
